# Medical student INtervention to promote effective nicotine dependence and tobacco HEalthcare (MIND-THE-GAP): single-centre feasibility randomised trial results

**DOI:** 10.1186/s12909-017-1069-y

**Published:** 2017-12-11

**Authors:** Anusha Kumar, Kenneth D. Ward, Lisa Mellon, Miriam Gunning, Sinead Stynes, Anne Hickey, Ronán Conroy, Shane MacSweeney, David Horan, Graduate Entry Programme 2014-18 Class, Alice Fox, Justin Greco, Sean Harris, Elise Halpern, Cong ( Johnny) Luo, Julie Peck, Alison Cameron-Vendrig, Adel Farah, Qasim Muhammad, Alexandra Mingay, Matthew Common, David Maj, Lorna Sampson Riden, Zhubene Mesbah, Carly Rabin, James Ulrich, Rehab Wali, Jodie Odame, Abhirami ( Anu) Ananth, Katie Dunleavy, Jessica Suddaby, Jared Sheridan, Kam Sing Ho, Samuel Mihalcioiu, Andrew Mikhail, Celia Fernandez, Farah Adamali, Samantha Steinmetz-Wood, Michael Strader, Madeleine Sugar, Humraaz Samra, Rachel Dharamshi, Camille Tastenhoye, Orla Fitzpatrick, Catriona Harte Hayes, Lisa Ann Lawlor, David Horan, Ahmed F. Hawwa, Sarah Lally, William Powell-Brett, Corry James O’Sullivan, Christian Ezeani, Andrew Redmond, Jessica Ennis, Florike Sheehan, Caroline Louise Anne O’Leary, Aoife Adelaide Gordon, Fearghal Martin Donnelly, Claire Drumm, Shane Karl Farrington, Aidan Alfred Mc Iver Bundy, Caroline Waddacor, Caroline Treacy, Sean Callinan, Gemma Wright Ballester, Dominic O’Dowd, Shona Reynolds, Francis Prendergast, Saoirse Ni Bhaoill, Liam Cormican, Seamus Sreenan, Frank Doyle

**Affiliations:** 1 Department of Medicine, Royal College of Surgeons in Ireland, Dublin 2, Ireland; 2 Division of Social and Behavioral Sciences, School of Public Health, University of Memphis, Memphis, USA; 3 Division of Population Health Sciences (Psychology), Royal College of Surgeons in Ireland, Dublin 2, Ireland; 4 Department of Health Promotion & Improvement, Health & Wellbeing Division, Health Services Executive, Blanchardstown, Dublin 15 Ireland; 5 Division of Population Health Sciences (Epidemiology & Public Health Medicine), Royal College of Surgeons in Ireland, Dublin 2, Ireland; 6 Department of Respiratory Medicine, Connolly Hospital, Dublin, 15 Ireland; 7 Director, Graduate Entry Medicine, Royal College of Surgeons in Ireland, Dublin 2, Ireland

**Keywords:** Smoking, Randomised trial, Medical students, Feasibility study, Mixed methods

## Abstract

**Background:**

Although brief cessation advice from healthcare professionals increases quit rates, smokers typically do not get this advice during hospitalisation, possibly due to resource issues, lack of training and professionals’ own attitudes to providing such counselling. Medical students are a potentially untapped resource who could deliver cessation counselling, while upskilling themselves and changing their own attitudes to delivering such advice in the future; however, no studies have investigated this. We aimed to determine if brief student-led counselling could enhance motivation to quit and smoking cessation behaviours among hospitalised patients.

**Methods:**

A mixed-methods, 2-arm pilot feasibility randomised controlled trial with qualitative process evaluation enrolled 67 hospitalised adult smokers, who were recruited and randomized to receive a brief medical student-delivered cessation intervention (*n* = 33) or usual care (*n* = 34); 61 medical students received standardised cessation training and 33 were randomly assigned to provide a brief in-hospital consultation and follow-up support by phone or in-person one week post-discharge. Telephone follow-up at 3- and 6-months assessed scores on the Motivation to Stop Smoking Scale (MTSS; primary outcome) and several other outcomes, including 7-day point prevalent abstinence, quit attempts, use of cessation medication, and ratings of student’s knowledge and efficacy. Data were analysed as intention to treat (ITT) using penalised imputation, per protocol, and random effects repeated measures. Focus group interviews were conducted with students post-intervention to elicit their views on the training and intervention process.

**Results:**

Analyses for primary and most secondary outcomes favoured the intervention group, although results were not statistically significant. Point prevalence abstinence rates were significantly higher for the intervention group during follow-up for all analyses except 6-month ITT analysis. Fidelity was variable. Patients rated students as being “very” knowledgeable about quitting and “somewhat” helpful. Qualitative results showed students were glad to deliver the intervention; were critical of current cessation care; felt constrained by their inability to prescribe cessation medications and wanted to include cessation and other behavioural counselling in their normal history taking.

**Conclusions:**

It appears feasible for medical students to be smoking cessation interventionists during their training, although their fidelity to the intervention requires further investigation. A definitive trial is needed to determine if medical students are effective cessation counsellors and if student-led intervention could be tailored for other health behaviours.

**Trial registration:**

NCT02601599 (retrospectively registered 1 day after first participant recruited on November 3rd 2015).

## Background

Tobacco use is the leading global cause of preventable death [1]. In Ireland the prevalence of smoking is estimated to be 19.5% [2], with about 5200 people dying every year from smoking and related healthcare costs accounting for approximately €500 million [3]. Smoking is also a major cause of health inequality and cessation is therefore an important public health goal [1–3]. Cessation benefits are well established, with successfully quitting smoking shown to result in an increase in life expectancy of 10 years and more [1]. Cessation interventions are also one of the most cost-effective healthcare interventions [1, 4]. Smoking cessation is therefore a unique opportunity to promote health across all healthcare disciplines, with relatively fast and potentially profound public health implications [3, 4].

Several Cochrane reviews have demonstrated that provision of brief advice by healthcare professionals (HCPs) increases quit attempts and rates [5, 6]. Among hospitalized smokers, simple advice, along with nicotine replacement therapy and supportive contact for at least one month post-hospital discharge, increases likelihood of cessation by 37–54% [6]. Given the efficacy of such HCP interventions, implementation into routine clinical practice is now a key goal [6, 7].

Hospitalisation appears to be an ideal time to implement cessation interventions for several reasons: ability to identify smokers; availability of HCPs; increased patient receptivity/motivation; ability to manage withdrawal symptoms in-hospital; ability to facilitate follow-up [6–8]. Surveys in Dublin hospitals and internationally have suggested a consistent failure of HCPs to intervene in patients who smoke [9–15], with less than half of smokers being advised to quit across several studies. Similarly, low rates of use of smoking cessation medications are evident post-discharge, with, for example, only 4.5% of smokers using nicotine replacement therapy 3-months post-discharge in one recent Irish study [12]. Common barriers to delivering interventions have been reported by HCPs [4, 13, 14]:

- Lack of time
- Lack of knowledge, training and confidence
- Lack of a systematic approach across sites, wards or staff
- Staff beliefs that: cessation is someone else’s role; interventions are less effective than they actually are, or require more time than is necessary or available; smoking is merely a ‘habit’ not requiring treatment
- Perceived patient resistance, despite research suggesting the contrary.

Clearly, there is ample room for improvement in service provision, yet in the face of stringent service cutbacks [16], it may be that creative thinking is required to implement low-cost, scalable solutions to ensure that smokers receive such vital advice.

Students are the healthcare professionals of the future, and are potentially an untapped, cost-effective resource for providing brief intervention for health promotion, including smoking cessation. To date, literature has suggested that graduates do not feel prepared for the ‘real-life’ demands of clinical practice, including smoking cessation [17], and more intensive behaviour change teaching during residency has also been described as a potential solution [18]. Few studies have assessed students’ ability to deliver behaviour change skills [19]. One recent randomised study showed that students who interacted directly with patients had the highest objective structured clinical examination (OSCE) scores for smoking cessation skills, with role-playing next, but both conditions were significantly higher than scores for students who attended a lecture or underwent web-based training [20]. Results from a cluster randomised trial from 10 medical schools investigating the effect of a complex, web-based, role-playing and clerkship modelling tobacco education (intervention) with traditional tobacco education (control) were recently published [21]. Students in the intervention schools were not significantly more likely to get higher OSCE scores for tobacco care than in control schools, even if they did score higher on some important components of cessation care, such as suggesting behavioural strategies and using quitlines [21]. One potential reason for this negative outcome could be the lack of interaction with actual patients [20], and the lack of opportunity to use, and try out, one’s learning in real situations, as is deemed theoretically important [22].

In sum, medical students are potentially an untapped resource who could deliver cessation counselling, and it is possible that the attitudes of future doctors towards provision of vital smoking cessation advice could be positively influenced by specialist cessation training and interaction with smokers. However, it is unknown whether students could be effective interventionists. We explored these issues in a mixed-methods feasibility randomised trial.

### Aim and objectives

We conducted a mixed-methods study, utilizing a two-arm, parallel group randomised controlled trial (RCT) and a qualitative process improvement component, to evaluate the feasibility of a medical student-delivered intervention to enhance hospitalised patients’ motivation to quit smoking, their use of pharmacotherapy, and cessation outcomes.

The objectives were to:

- Determine the potential efficacy of student-delivered smoking cessation counselling on primary and secondary outcomes
- Obtain student evaluations of the training and intervention implementation

## Methods

We followed the CONSORT statement for RCT reporting [23] and the TIDieR [24, 25] statement for proper, detailed reporting of complex interventions.

### Participants

For the feasibility randomised trial, inclusion criteria were all identified inpatient smokers. Exclusion criteria were as follows:

- Advised by ward manager that patient was too unwell or cognitively impaired, or otherwise unsuitable
- Death during hospitalisation
- Receiving palliative care
- Under 18 years old
- To be transferred to another hospital
- Non-English-speaking
- Inpatient in psychiatric ward

The qualitative evaluation involved assessment of students’ perceptions of the training and delivery of the intervention – all students were invited to participate in focus groups. It was initially planned that all students would deliver the intervention, but too few smokers were recruited for this (see later).

### Interventions and procedures

Following the TIDieR checklist [25], the intervention was as follows:

1 brief name

Student-led brief smoking cessation advice.

2 why

The medical students delivered a brief (approximately 15 min) consultation with the patient that is based on principles of social cognitive theory [26] and motivational interviewing [27]. The goals of this consultation were to enhance the patient’s motivation and self-efficacy regarding quitting, educate the patient regarding effective behavioural and pharmacological cessation strategies, and to work with patients to devise strategies to help them refrain from smoking after discharge.

3 what

Materials consisted of a handbook for students provided during the motivational interviewing training, some student prompt sheets which also contained the outcomes of interest, along with videos of motivational interviewing interactions posted to the Royal College of Surgeons in Ireland (RCSI) virtual learning environment. These materials are available from the corresponding author on request. Patients in the intervention group who received a student consult and were receptive to using pharmacotherapy to aid cessation had a yellow-black coloured sticker placed by the student in the medical chart, stating:

A course of smoking cessation counselling has been delivered to this patient. The patient has requested a consultation with you to determine appropriateness of medication to aid abstinence. Please consider nicotine replacement therapy or other pharmacotherapy (if it is clinically appropriate at this point in time) to aid abstention. For your convenience, information about approved medications is attached.

Signed:...........................................................................

RCSI Graduate Medical Student

RCSI Graduate Entry Medicine Smoking Cessation Project.

4 procedures

To avoid treatment contamination, it was important that students did not deliver the intervention to the usual care group. Therefore, recruitment was conducted by an independent researcher, who visited wards to assess patient eligibility prior to student contact. AK, a clinical lecturer, took on the responsibility of patient recruitment and follow-up. AK recruited eligible patients, who were asked to participate in a program to help them quit smoking, which would involve receiving information about how to quit and possibly a consultation with a specially trained medical student. Informed written consent was obtained from participants. Relevant demographic (age, sex, education, living status, insurance status [Medical Cards are provided to people >70 and those with low incomes]) and clinical details (clinical history, Charlson Co-morbidity Index [28]), motivation to quit, smoking history (including Fagerstrom Test for Nicotine Dependence [29]) and attitudes towards quitting were obtained. AK then forwarded the patient contact details to the assigned student.

Student intervention was staggered over September 2015 to June 2016.

5 who provided

The intervention was to be provided by 61 s year Graduate Entry Medicine (GEM) students from the RCSI who received Brief Intervention for Smoking Cessation (BISC) training. Students had a variety of primary degrees before joining the GEM programme.

Pre-intervention medical student training took place on 14th September 2015, in three groups of ~20 students. Students received the one-day Health Services Executive (HSE) national standard BISC motivational interviewing-based cessation training available to all HSE staff (http://www.hse.ie/cessation/), which was delivered by experienced trainers. The HSE BISC is a short, patient-centred intervention which emphasises self-efficacy, personal responsibility for change, information giving and details of resources available to support change, including pharmacotherapy education. BISC uses the 5As approach (Ask, Advise, Assess, Assist, Arrange follow-up) as its central framework.

6 how

Students individually delivered the intervention face-to-face with individual patients.

7 where

The intervention took place on the inpatient wards of Connolly Hospital (www.connollyhospital.ie), which is an RCSI-affiliated teaching hospital in Dublin, Ireland.

8 when and how much

Once notified about a recruited participant the student then went to the relevant ward and counselled the patient within the next 2–3 days, including obtaining data for the outcomes of interest. This typically lasted ~15–20 min. Students also re-contacted the smoker at 1-week post-discharge via telephone or personal follow-up, in order to provide further support. This typically lasted ~10 min.

9 tailoring

Tailoring to an individual smoker’s needs was emphasised during training. Students were encouraged to elicit patients’ personal barriers to cessation, and discuss how to overcome these.

10 modifications

No modifications were made during the study.

11, 12 Fidelity.

Planned fidelity check was asking students to complete details on the student prompt sheet. Actual fidelity was assessed by determining the completeness of the student prompts, i.e. the proportion of answers provided by the students on their guide questionnaire for performing the intervention. We did not have the resources to follow more intensive fidelity procedures (e.g. observations/recording of individual sessions).

### Comparator (usual care) group

This group received whatever treatment happened as a normal part of the inpatient stay (e.g. a visit from the smoking cessation officer). In usual care if patients request cessation services, physicians may prescribe pharmacotherapy and/or refer them to the hospital cessation officer or the national quitline (www.quit.ie, Freephone 1800 201,203).

### Outcomes

The primary outcome was change in motivation to quit, as assessed by the Motivation to Stop Smoking Scale (MTSS) [30], which is a single-item assessment of self-rated motivation to quit smoking, using a 7-point scale (“I don’t want to stop smoking” through “I really want to stop smoking and intend to in the next month”). The MTSS has been shown to accurately predict the odds of making a quit attempt among smokers [30]. Those who had already quit during follow-up were allocated the maximum score of 7.

Secondary outcomes included the following:

- the proportion of patients who receive a prescription for a cessation medication at the time of discharge, assessed via medical chart audit;
- self-reported 7-day point prevalent abstinence rates assessed at both 3- and 6-months
- the proportion of patients who reported any use of a prescribed or over-the-counter cessation medication, of an approved cessation pharmacotherapy, including nicotine patch, gum, lozenge, inhaler, mouth spray, varenicline, or buproprion at 3- and 6-months discharge.

The proportion of physicians prescribing cessation medications was initially listed as a secondary outcome, but this information was not recorded due to resource constraints.

Other outcome measures were:

- patient-reported effectiveness of the medical students as interventionists (i.e., “How helpful was the support that you received from the medical student?” [not at all, a little bit, somewhat, quite a bit, or very much] [31]; and “How knowledgeable was the medical student about quitting smoking?” [not at all, a little bit, somewhat, quite a bit, or very much]);
- Quit attempts
- Receiving professional advice regarding quitting

Primary, secondary and other outcomes were assessed at 3- and 6-months post-discharge, via telephone.

### Sample size

Our outcomes were intended to provide critical feasibility data needed to inform a future, large-scale RCT of the effectiveness of medical students in promoting use of cessation pharmacotherapy and improving long-term cessation outcomes. Therefore, the power to detect changes was considered less important than insights from the process evaluation. We aimed to recruit a total of 180 smokers and predicted a 25% loss to follow-up, leaving 135 smokers available for per-protocol analysis. This would allow each student to counsel 1–2 smokers each.

All students were invited to the process evaluation and 25 (14 women) participated in three focus group discussions with FD, KW (2 focus groups) and FD, LM (1 focus group). These took place in May and June 2016. Interviews with students determined their thoughts on how the intervention went, their attitudes to cessation counselling, and what could be done to adapt/improve the programme.

### Randomization and allocation concealment

Patients were block-randomised using the user-written Stata *ralloc* command [32], with random block sizes ranging from 2 to 10 [33], by FD. Student interventionists were randomly allocated, without replacement, to each intervention patient in turn. We planned that each student would deliver one intervention before any student delivered two interventions. Once patients were recruited, AK emailed the participant number to FD, who provided the group allocation.

### Blinding

AK was blind to group allocation when recruiting the patients. No other blinding was implemented.

### Analysis methods

Group differences at baseline were not assessed as per CONSORT and recent guidelines [23, 34]. Repeated measures analysis of variance assessed the primary outcome. Data were analysed both on an intention-to-treat (ITT) using penalized imputation (i.e., assuming that patients who did not provide follow-up data, or who died during follow-up, did not use cessation pharmacotherapy or were not abstinent, or assuming no change in motivation level from baseline), and as per protocol analysis due to concerns that penalized imputation may not necessarily be conservative with regard to treatment effects [35–37]. To maximise the repeated measures data, and to account for the different interventionists and concerns over just using traditional analyses [37], we also analysed outcomes using random effects repeated measures panel modelling (xt commands) in Stata 14.2. Therefore, (repeated measures) linear/logistic regression was used to predict outcomes at both 3- and 6-months, using all available (per protocol) data, with missing data considered missing at random [37]. Adjustment for multiple comparisons or covariates was not made as this is a feasibility trial.

Qualitative data were analysed using a thematic approach by two researchers (FD and SMcS) [38].

## Results

### Quantitative results

#### Participants

**Fig. 1:**
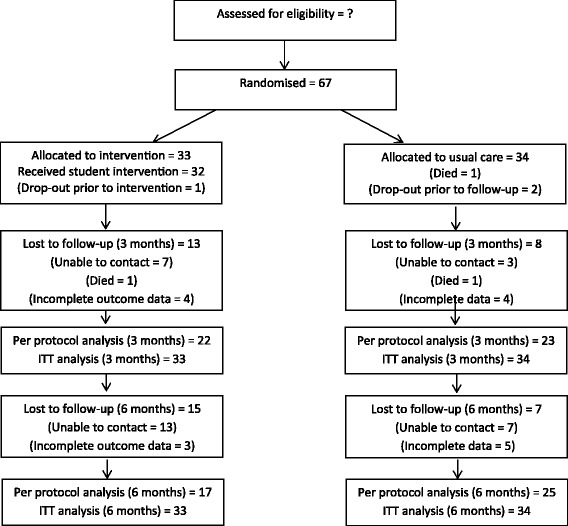
Participant flowchart

The participant flowchart is highlighted in Fig. 1. Unfortunately the number of patients who were approached to participate was not recorded, and therefore the recruitment rate is unavailable.

Only 67 participants were recruited, instead of the targeted 180. Several participants were lost to follow-up, or withdrew, at different stages. Participant profile by group is shown in Table 1.

**Table 1:** Sample profile

	Intervention (*n* = 33)	Control (*n* = 34)
Age, mean (SD)	57.5 (14.6)	59.7 (13.4)
Men	66.7%	58.8%
Education: 3rd level	24.24%	20.6%
Married/cohabiting	51.5%	52.9%
Lives with others	78.8%	85.3%
Lives with smoker	24.2%	35.3%
Insurance		
Medical card (low income or those >70 years)	48.5%	55.9%
None	39.4%	32.4%
Private/Other	12.1%	11.8%
Days since admission, median	6.5 (IQR 3–11)	4.5 (IQR 2–7)
Elective surgical admission	18.2%	17.6%
Emergency admission	84.8%	82.4%
Charlson Co-morbidity Index, mean	2.42 (1.97)	2.56 (1.89)
HCP quit advice in past year	39.4%	50%
Quitting discussed during admission	27.3%	32.4%
Smoking status recorded in chart	54.6%	67.7%
Like to receive advice	100%	100%
Cigarette smoker (vs. cigar/pipe)	93.9%	97.1%
No. of cigs per day, mean	17.6 (11.1)	17.2 (13.0)
Current smoking		
Smokes every day	39.4%	58.8%
Smokes some days	9.1%	–
Not smoking in hospital	51.5%	41.2%
Years smoked, mean	38.7 (18.0)	42.3 (14.6)
Fagerstrom (FTND), mean	2.63 (1.39)	2.87 (1.45)
Quit 1 or more days in past year	45.5%	33.3%
No. quit attempts in past year, mean	2.43 (1.67)	2.36 (2.73)
MTSS, mean	4.97 (1.36)	4.91 (1.42)
*Attitudes to quitting:* Do you think that if you gave up smoking…		
…your health would improve in the short-term:		
Yes	75.8%	76.4%
Unsure	21.2%	17.7%
No	3.0%	5.9%
…your health would benefit in the long-term:		
Yes	93.9%	73.5%
Unsure	6.1%	14.7%
No	0%	11.8%
…you would put on weight:		
Yes	18.2%	14.7%
Unsure	69.7%	58.8%
No	12.1%	26.5%
…it would be harder to handle stress in your life:		
Yes	34.6%	70.6%
Unsure	12.1%	5.9%
No	51.5%	23.5%
…you would feel you had done something worthwhile:		
Yes	87.9%	73.5%
Unsure	3.0%	17.7%
No	9.1%	8.8%

IQR (interquartile range); *p < .05, **p < .01, ***p < .001

Overall, the samples appear to be well balanced. There were different attitudes between intervention and usual care groups, with the intervention group somewhat more likely to endorse the statement that their health would benefit in the long term if they quit, while the usual care group were more likely to believe that it would be harder to handle stress if they quit.

#### Numbers analysed

All analysis was by original assigned groups, with full data used for ITT analysis. See Fig. 1 and Table 2 for the numbers analysed for per-protocol analysis.

**Table 2:** Analysis of trial outcomes assessed at two or more time-points

	3 months (n)		6 months		Per protocol analysis: 3-month	ITT analysis: 3-month	Per protocol analysis: 6-month	ITT analysis 6-month	Repeated measures (random effects analyses)
	Intervention	Usual Care	Intervention	Usual Care					
Primary outcome:									β = .57 (−.03 to
MTSS; mean (SD), number(n)	5.43 (1.36) (*n* = 21)	4.57 (1.73) (*n* = 23)	5.65 (1.46) (*n* = 17)	4.27 (2.21) (*n* = 22)	F = 0.66, df = 4, *p* = 0.619	F = 0.59, df = 4, *p* = 0.671	F = 1.42, df = 3, *p* = 0.239	F = 1.53, df = 3, *p* = 0.207	1.18), *p* = 0.064
Secondary outcomes:									
Reported use of cessation medications	PP = 60% (*n* = 20) ITT = 18.2%	PP = 42.9% (n = 21) ITT = 14.7%	PP = 73.3% (*n* = 15) ITT = 9.09%	PP = 44% (*n* = 25) ITT = 17.7%	OR = 2.0 (0.58 to 6.94), *p* = 0.275	OR = 1.29 (.35 to 4.7), *p* = .701	OR = 3.5 (0.87 to 14.1), *p* = .077	OR = 0.47 (.10 to 2.04) *p* = .312	OR = 22.4 (0.31 to 1626.5), *p* = 0.155
7-day point prevalent abstinence rates	PP = 22.7% (n = 22) ITT = 27.3%	PP = 4.4% (n = 23) ITT = 5.9%	PP = 58.8% (n = 17) ITT = 30.3%	PP = 20.8% (*n* = 24) ITT = 14.7%	OR = 6.47 (.69 to 60.7), *p* = .102	OR = 6.0 (1.18 to 30.3), *p* = 0.030*	OR = 5.43 (1.37 to 21.6), *p* = .016*	OR = 2.52 (0.76 to 8.41), *p* = 0.132	OR = 7.2 (1.10 to 47.3), *p* = .040*
Other outcomes:									
Quit attempts (any = 1, none = 0)	PP = 50% (n = 22) ITT = 42.4%	PP = 39.1% (n = 23) ITT = 29.4%	PP = 70.6% (n = 17) ITT = 36.4%	PP = 41.7% (n = 24) ITT = 29.4%	OR = 1.56 (.48 to 5.08), *p* = .464	OR = 1.77 (.64 to 4.86), *p* = .269	OR = 3.36 (.90 to 12.6), *p* = .072	OR = 1.37 (.49 to 3.81), *p* = .545	OR = 2.1 (.89 to 5.0), *p* = .089
Receipt of professional quit advice	PP = 40% (n = 20) ITT = 24.4%	PP = 27.3% (n = 22) ITT = 17.7%	PP = 46.7% (n = 15) ITT = 21.2%	PP = 42.1% (*n* = 19) ITT = 23.5%	OR = 1.78 (.48 to 6.5), *p* = .384	OR = 1.49 (.46 to 4.9), *p* = 0.508	OR = 1.2 (.31 to 4.7), *p* = .790	OR = 0.875 (0.28 to 2.77), *p* = .820	OR = 1.56 (.52 to 4.7), *p* = .429

*p < .05; Inter = intervention group; ITT = intention to treat analysis (denominator is 33 and 34 respectively for intervention and usual care – see Fig. 1); PP = per protocol analysis (denominator changes due to missing data for each outcome)

#### Outcomes

Results for repeated primary and secondary outcomes that were repeated over follow-up are reported in Table 2. Mean MTSS scores were as follows: baseline (intervention 4.97 [SD 1.36]; usual care 4.91 [SD 1.42]) and 1-week (intervention 5.4 [1.27]; usual care 4.59 [1.53]) – see Table 2 for 3- and 6-month data.

Of note, although most analyses are not statistically significant, the effect size points in the direction that favours the intervention for almost all of the per protocol, ITT and repeated measures analyses. The exceptions are ITT 6-month analyses of reported use of cessation medications and receipt of professional cessation advice, which have ORs < 1, favouring the usual care group. Point prevalence abstinence rates were consistently significantly higher in the intervention group, with only the 6-month ITT analysis non-significant.

Being prescribed cessation medication on discharge was reported by 15.2% of intervention, and 17.7% of usual care patients (OR = 0.83, 95% CI 0.23 to 3.0, *p* = .783).

Intervention patients rated students as being “very” knowledgeable about quitting (*n* = 27, mean = 4.52 [SD = .77]), and “somewhat” helpful (*n* = 29, mean = 3.37 [SD = 1.47] out of 5).

#### Fidelity

Student prompt items were completed for 39.4% (reason for confidence score) to 57.6% (reason for motivation score; addition of chart sticker).

#### Qualitative results: “…a lot of the time, we are just furniture”

Three focus groups were conducted with a total of 25 student participants (11 men, 14 women). The first and third focus groups were a mix of those who had and those who had not delivered the intervention, whereas the second focus group comprised only students who had not delivered the intervention. All participants had received the 1-day BISC training.

Four main themes were obtained from the analysis (Table 3). These comprised reflections on the overall process as a positive learning experience, being critical of current cessation care, feeling constrained by their role as students in training, and proposing solutions going forward, such as generalising the skills acquired and implementing these at opportune moments.

**Table 3:** Themes from qualitative analysis

Training and intervention as a positive experience	“I had a very positive experience as well because it was pre-decided that the people we were going up to in order to ask if they wanted help, had already agreed that they did want help. So I think if we were just going up to known smokers who weren’t at least open to the idea, I would say you could get a few negative experiences as well.” (Focus group [FG] 1)
	“It felt more like legitimate patient care, I would say, than me going in and practicing an exam for me. As opposed to trying to find out what’s wrong with the patient. So it felt more needed for the patient and myself, as opposed to just selfishly practicing an exam on a patient” (FG1)
	“Interviewer: Could I ask – would anybody like to do it again? Student(s): Yeah. Student 1: As I was saying earlier about it - I think, as a medical student, I think it’s good for us to get experience talking to patients because while they’re there for our benefit, we can actually benefit them at the stage we’re at. I think that’s very good.” (FG1)
Critical of current smoking care	“My patient was on Nicorette patches. And on the week follow up he was basically discharged without a refill prescription for Nicorette patches. So that struck me. I’m not sure if the smoking cessation officer or nurse saw or not, or if another doctor would think to prescribe that. But it just shows me how it really is not a priority at all. That’s what it showed me.” (FG1)
	“No, they rarely talk about smoking. I had maybe one experience. It wasn’t with smoking; it was part of the visit that they just counselled them as smoking. They just said “you need to stop smoking.” Those exact words.” (FG2)
	Interviewer: “…do you think that those doctors have time to do this the way you guys were trained? (some disagreement among students followed) Student 3: Oh in the actual clinic? Yeah, that’s a different story. I was thinking more of the patients in their care in the wards. I think they would definitely have the time. A lot of times, let’s say the SHO or the registrar is waiting for the consultant to show up. Before that they could definitely spend ten or fifteen minutes to add this on, because they pre-wrap anyway, so. Interviewer: Do you think you guys could help out in a clinic, in a setting like that? Student 3: Yeah absolutely. I mean in a clinic, I don’t know about you guys, a lot of the time we are just furniture.” (FG1)
Frustration from constraints/ difficulties	“Mine didn’t even know that there was such a thing as nicotine replacement therapy and that threw me off. And then I knew I wasn’t technically supposed to talk to him about it. But I sort of did and then I found out later that the smoking cessation counsellor hadn’t gotten to him because he’d been distracted.” (FG3)
	“…. Like, if you’re fully trained you can say there are other things to help you other than smoking, right? But we’re not really at a stage to start prescribing Nicotine replacement.” (FG3)
Solutions/ improvements for the future	“… we are encouraged by all of the consultants to take a thorough history on the wards. So I don’t think we should just go and talk to them only about smoking. It’s much smoother if you just take a history of the patient, and then talk a little bit about smoking. And in that way, you could be like “hey I heard someone talked to you about smoking. How do you feel about that now?” Then you’re giving them a day to think about it, instead of on the spot kind of motivation. So they have a chance to go do some other stuff.” (FG3)
	“I think that maybe including a demonstration in the tutorial of how you incorporate smoking cessation into taking a history. So not just on its own but a complete demonstration putting it together with a complete history and seeing how it fits in with all of that.” (FG 3)
	“I think that the forms do a perfectly fine job of assessing how motivated and confident they are. But if it’s more to focus on what would actually benefit the patient, I think they are too limited in evaluating and motivating them because it’s only three quick questions.” (FG1)

There were also disagreements among the students on various issues. For example, some students disagreed to the extent to which this was legitimate patient care, or just training (Table 3). Furthermore, the use of the standardised sheets was seen as a negative in some instances (see Table 3), but as a positive if patients were not that talkative at that time. Patient follow-up at one week was also reported as being difficult, due to availability and timing issues, so students reported trying to do this on multiple occasions.

Notably, students in focus group 2, who had not delivered the intervention, were more likely to think that time was an issue for delivering the intervention, and were less confident about the overall process.

Finally, students also suggested perhaps finding patients themselves, not having them pre-screened, and incorporating cessation counselling into standard history taking (Table 3).

## Discussion

We report the first pilot feasibility randomised trial of a medical student-delivered smoking cessation intervention [19]. Results showed that medical students could be effective smoking cessation interventionists, although this would have to be established in an adequately powered study. Process evaluation highlighted strengths and weaknesses of the programme, but overall it was received as an educationally positive intervention.

### Evaluation of feasibility findings

As this was primarily a pilot feasibility study, appropriate dimensions of study implementation deserve comment [39, 40]. *Acceptability* was high: patients rated students as being helpful and knowledgeable, while students appreciated the training and the opportunity to practice these skills in a real world setting, with some even stating that it felt like real patient care. *Demand* for the intervention can be thought of as high in terms of the need for cessation counselling in current settings [9, 11, 12], but low in terms of the ability to recruit and retain patients, since we recruited less than half the targeted numbers and there was substantial loss to follow-up. However, this is not uncommon in smoking cessation trials (e.g. [37]). Unfortunately we did not have a dedicated staff member who could spend more time on recruitment, and this may have impacted on our overall numbers, and we do not have an estimate of refusal rates as the numbers approached were not recorded. *Implementation* of the intervention was possible – students attended training, which was completed in one day, and then were randomly assigned to counsel smokers. While there was no difficulty with this aspect of the design, we have limited details of the fidelity of delivery of the cessation counselling – just because students completed the items does not mean that these were delivered accordingly, and vice versa, although the proportions of completed student prompt items was variable. We deemed it more important that students had the opportunity to complete a brief intervention and not be burdened completing detailed fidelity forms which would be of high scientific value, but perhaps of lower educational value. Indeed, some student reflections on the prompt sheets suggested that these were of limited value, perhaps because they did not follow the flow of the conversation with patients. Future research should investigate the level of fidelity to motivational interviewing and recommended cessation techniques, by observing or recording individual sessions.

The intervention is *practical*, in that it was designed to be delivered to overcome resource constraints, albeit that it requires resources to recruit participants. Future research could investigate how such an intervention would be received if students were to find potential participants themselves, rather than have these recruited for them. *Adaption* was not assessed in this study, although by its nature motivational interviewing is designed to be adapted to individual patients. We did not change procedures for the study, and this was performed in one centre only, so it is currently unclear how it could be satisfactorily implemented in other settings, with curriculum restraints in larger classes, or with typically younger direct entry medical students, or other healthcare professional students etc. *Integration* was achieved relatively easily with the enthusiastic support of the HSE, GEM and hospital staff. *Expansion* can only be investigated in future work. *Limited efficacy testing* will be discussed next*.*

### Study outcomes

No significant difference in the study primary outcome, motivation to quit over time, was seen. However, motivation scores were somewhat higher in the intervention group during follow-up, suggesting some limited effect that ultimately the study was not powered to detect due to the lower than expected recruitment. It may also be that the MTSS scale was not sensitive enough to detect changes in motivation to quit, and future work should use other, more established motivation tools. This is especially pertinent as it is unclear how to score the MTSS when a person has already quit – we imputed the highest score, but it is probable that alternative higher score (e.g. a score of 8) for quitters would have yielded a statistically significant result, given the differences in quit rates seen between groups.

While most results for the secondary outcomes were also non-significant, almost all results favoured the intervention group. This highlights the potential of this intervention for enhancing quality of smoking cessation care in hospital settings, although a definitive trial is needed to determine this. One outcome that was consistently significantly higher in the intervention group was quit rates. This was despite the lack of difference in motivation to quit, which was the theoretical mediator of quitting. The effect sizes were higher than in other smoking cessation randomised trials [6], however, and this may reflect methodological issues and a lower than expected quit rate in the usual care group, leading to overestimates of the effects. Caution should also be used considering that multiple tests were conducted. Furthermore, there were more positive attitudes towards quitting seen in the intervention group at baseline – it is possible that these attitudes sustained quit behaviours beyond the student intervention and that these account for the significant findings and the positive trends for other secondary outcomes.

### Educational aspects

Practical training, such as that provided here, fits with the Kirpatrick model [22] – students reacted favourably, acquired skills and knowledge, (some) applied their skills, and outcomes (i.e. enhanced quality of cessation care) appear to have been met, at least in terms of trends in the data. As an educational intervention, it was notable that the student evaluations of the programme were much more positive when they had actually applied their training with a patient, allowing them to demonstrate integrative learning, which is usually appreciated by students [22, 41, 42], and may lead to better preparedness for practice post-graduation [17, 18]. One illustration of integrative learning was that students proposed potential solutions to the issues with implementing this counselling – e.g. incorporating this into current history taking. Furthermore, training similar to that provided here, can lead to higher OSCE scores than simple web-based or lecture-based tuition [20]. Applying the training with real patients also overcomes issues of low student engagement, as has been reported in other behaviour change training [43]. While feedback was positive, it is unknown whether this training will have a longer-term impact on students’ attitudes towards cessation counselling, or indeed future behaviour as clinicians, but at least students should feel better prepared for real work needs [17]. This needs to be investigated in future work. It is possible that at least some of the reported barriers to provision of smoking cessation care, as outlined earlier (e.g. perceived lack of time and lack of training), may be overcome by the programme we outline. It was notable that students who had delivered the intervention were more likely to state that doctors did actually have time to deliver cessation counselling, and were more confident about the overall process, when compared to students who did not counsel patients. This fits with expectations from educational and behavioural science theory: that those who get to practice their learned skills have enhanced self-efficacy to deliver this in the future [22, 26, 27].

### Ethical issues

This study posed some ethical questions that deserve comment. The study was approved by two Research Ethics Committees, with the recommendation that as students were interventionists, they also become study investigators. Students were also insured to provide this cessation counselling. We also agreed a protocol where students would not advise on cessation medication dosages as this was beyond their training – which students found frustrating, as outlined above. Placing coloured stickers in the medical charts indicated without ambiguity that such cessation advice came from students.

### Limitations

There are several potential study limitations. First, although our ultimate goal is to increase smoking cessation rates, our primary outcome in this study was a subjective variable (motivation to quit), given the pilot nature of the study and the importance of motivation to quit as a mediator of cessation [30, 44]. However, the primary outcome measure, the MTSS, strongly predicts the odds of making a quit attempt [30]. Second, although we did assess smoking cessation as a secondary outcome, we relied on self-report due to resource limitations. Some degree of false positive reporting is likely, although differential bias is mitigated because both intervention groups knew they were participating in a smoking cessation intervention and cessation was assessed using an identical protocol. Third, like most behavioral interventions, it was not possible to blind participants or interventionists to allocation assignment. Fourth, there may be variation in the delivery of care among students, and with only one intervention each it is likely that students may actually improve with more practice, but this cannot be assessed in the current research. We attempted to account for this between-student variability with the random effects modelling, but estimates may have been affected by small numbers. Fifth, other changes in staffing and/or policy may confound any demonstrated effects – for example, an increase in hours or numbers of smoking cessation officer(s). As with most RCTs, the results may not be generalizable to the wider population of smokers – particularly, smokers who participate in a cessation trial are already motivated to quit – it is unknown how the students would be received by patients who have not agreed to participate. Sixth, we were not powered to obtain statistically significant differences, even though the changes here are arguably clinically relevant. Lastly, we do not contend that students could become expert motivational interviewers after a single day’s training and practice with one patient (sometimes with a wide timeframe between each). However, a single day’s training is all that could feasibly be delivered in the current, full curriculum. Furthermore, we hope that the present study is a first step towards longer term attitudinal and behaviour change in RCSI graduates with regard to smoking cessation counselling. Even with these limitations, upskilling students in smoking cessation counselling, enhancing their self-efficacy to deliver such counselling and making smoking cessation a priority for the next generation of doctors, is still a very worthwhile endeavour.

## Conclusions

It appears feasible for medical students to be cessation interventionists during their training, and this training and intervention practice was appreciated by students. A definitive trial is needed to determine levels of intervention fidelity, whether medical students are effective cessation counsellors and if student-led intervention could be trialled for other health behaviours. We believe, based on this study, that all medical students should be given training in brief motivational interviewing, and have an opportunity to apply these skills when discussing smoking cessation with an actual patient.

## Abbreviations

5As: Ask, Advise, Assess, Assist, Arrange follow-up
BISC: Brief intervention for smoking cessation
CCI: Charlson Co-morbidity Index
FG: Focus group
FTND: Fagerstrom Test for Nicotine Dependence
GEM: Graduate Entry Medicine
HCP: Healthcare professional
HSE: Health Services Executive
ITT: Intention to treat
MTSS: Motivation to stop smoking scale
OSCE: Objective structured clinical examination
PP: Per protocol
RCSI: Royal College of Surgeons in Ireland
SHO: Senior House Officer
